# Use of Social Networks in the Context of the Dietitian’s Practice in Brazil and Changes During the COVID-19 Pandemic: Exploratory Study

**DOI:** 10.2196/31533

**Published:** 2022-02-25

**Authors:** Jackson Sbardelotto, Bárbara Birck Martins, Caroline Buss

**Affiliations:** 1 Nutrition Course Federal University of Health Sciences of Porto Alegre Porto Alegre Brazil; 2 Department of Nutrition Graduate Study Program in Health Sciences Federal University of Health Sciences of Porto Alegre Porto Alegre Brazil

**Keywords:** dietitian, social networks, nutrition, health communication, COVID-19, social media, Brazil, perception, health information, usage, behavior

## Abstract

**Background:**

Social networks have been pointed out as 1 of the greatest means of spreading information. A large part of the population is already present on these platforms, looking up subjects such as health, nutrition, and food. To reach this audience, it may be important for dietitians to explore social networks. However, there is a gap in scientific studies on exploring the ways in which these platforms are used by dietitians in Brazil, and the roles they play in the profession have not been well defined.

**Objective:**

This study aims to describe the roles that social networks play in dietitians' practice in Brazil and their mode of use of social networks. This study also aims to identify professionals’ perceptions and opinions regarding the use of these tools, as well as changes in behavior on social network usage caused by the COVID-19 pandemic.

**Methods:**

We carried out a quantitative cross-sectional study, collecting data through an online questionnaire, submitted between October 2020 and January 2021 to dietitians registered on the Federal Council of Dietitians. All participants included in the study answered questions about the use of social networks in their professional context.

**Results:**

In total, 264 (91.7%) of the 288 participants reported using social networks for professional practice. Instagram was the social network most often used by professionals (224/264, 84.8%). Dietitians (N=288) related to the use of social networks (always to almost always) for sharing information about their services (n=114-72 [39.6%-25%], respectively), following the work of other dietitians (n=172-64 [59.7%-22.2%], respectively), and writing about topics related to food and nutrition (n=166-53 [57.6%-18.4%], respectively). The roles played by social networks in the professional context of dietitians were attracting more clients (210/289, 72.7%) and keeping in touch with them (195/289, 67.5%). Furthermore, 227 (78.5%) of the 289 dietitians strongly agreed that social networks are good tools to promote their services. During the COVID-19 pandemic, 216 (74.7%) of the 289 participants noticed changes in their behavior, feelings, or beliefs on the use of social networks related to professional practice, and 149 (51.6%) have increased the frequency of sharing information about nutrition and health in general on social networks.

**Conclusions:**

The main roles of social networks in the professional context of dietitians are to attract clients and to facilitate the contact between professional and client. The modes of use reported by the professionals included sharing information about their services, following the work of professional colleagues, and writing about topics related to nutrition. Most of them reported believing that social networks are an effective way to disseminate their services. Moreover, most professionals claimed to have noticed changes in their behaviors or beliefs on social media during the COVID-19 pandemic.

## Introduction

Social networks have been pointed out as 1 of the greatest means of spreading information, with 4.2 billion active users in the world, which corresponds to 53.6% of the world population [1]. In 2019, in a prepandemic scenario, the percentage of active users around the world was 42%, representing about 3 billion users [2]. The year 2020 marked the biggest growth in the number of users in the past 3 years. The internet has become 1 of the main sources for seeking information about health-related matters, especially with the restriction of face-to-face contact due to the COVID-19 pandemic [3].

About 72% of adult internet users search for information about diagnoses, treatments, and reports of people in the same health condition [4]. Social networks can have many benefits for health professionals’ practice, by providing greater possibility to interact, share experiences, and communicate in real time with other health professionals as well as their clients/patients [5]. One health-related topic with high interest on social networks is regarding nutrition and healthy eating. Evidence shows that possible impacts of social networks can be related to facilitating eating behavior changes, as they allow users to acquire some knowledge and understanding about healthy eating [6].

Social networks have reach to a large audience and are low-cost tools. Therefore, social networks are considered tools with potential to offer great benefits to dietitians. They can assist professionals in their online nutritional interventions, disseminating evidence-based information, and can work as an extension of their service [7,8]. A large part of the population is already present on these platforms, looking up subjects such as health, nutrition, and food [9]. Many people started to do so when there was a restriction of face-to-face consultations during the period of social isolation due to the COVID-19 pandemic. At the same time, researchers are using social media to share recommendations and decisions being made in times of the COVID-19 pandemic. In addition, dietitians have had to shift from in-person client interactions to telenutrition consultations [3,10].

The estimated average time spent on social media per user around the world is currently 2 hours and 25 minutes a day [1]. Therefore, to reach this audience, it may be important for professionals to explore social networks to provide adequate information about nutrition and food, to implement the practice of educational actions, and to offer nutrition services. In Brazil, the average time spent per day by users of social networks is 3 hours and 42 minutes. Brazil is in third place in this global ranking [1]. Thus, conducting studies on this subject in the country is valid and necessary to make possible a better understanding of the role of social networks in the profession and, later, to find opportunities of its use.

In the light of the increasing use of social media around the world among dietitians and the general population, Dumas et al [11] designed a review to map existing evidence on the effects of social media on nutritional practice. The authors found 4 interventional studies with dietitians and social media users. From the users' perspective, online interaction with professionals can be favorable for changing behavior. However, further studies isolating variables will be necessary to measure the effectiveness of networks in this process. We also identified studies with expert opinions about the behavior of professionals on social networks, dealing with advantages and disadvantages of the use of networks in the profession and guides on ethical conduct on digital platforms [6,7,12]. Few of these have been studied so far (ie, discussion forums, blogs, and Facebook).

Saboia et al [13] observed the influences on the eating behavior of the followers of dietitians and nongraduates on Instagram in Brazil and Portugal. They were able to describe some usage characteristics of the professionals and how they interact with the public. However, it is necessary to further explore the use of networks in the profession to identify their modes of use.

Another review conducted by Saboia et al [14] also evidenced the lack of studies that can describe how dietitians are using social networks. In a 2020 publication [15], the same authors designed a questionnaire to understand the dynamics of social media use by dietitians for future application with dietitians in Portugal.

Given the low number of studies that have actually explored the ways that dietitians use social media, and how the COVID-19 pandemic may have altered such use, we developed this study. The aim of this study was to identify how dietitians in Brazil use social networks and to describe the professionals' perceptions and opinions on the use of these tools, as well as changes in behavior on social network usage due to the COVID-19 pandemic.

## Methods

### Study Design

We conducted a quantitative cross-sectional study, collecting data through an online questionnaire, submitted between October 2020 and January 2021. People participated in the research only after reading and being aware of the free and informed consent terms, which provided information about the nature of the study.

### Ethics Approval

The research ethics committee of the Federal University of Health Sciences of Porto Alegre approved the study (#3981023).

### Sample and Participants

According to data from the Federal Council of Dietitians (FCD), there were 150,892 active dietitians in Brazil in 2019 [16]. Adopting a conservative approach to estimating the prevalence of the use of social media by dietitians, a ratio of 50% was used to maximize the sample size. A sampling error of 6% and a 95% CI were adopted. The minimum number of responses for the sample to be representative of this population was calculated as 267 participants.

The criterion for being included as participants was to be registered with the Regional Council of Dietitians (RCD). We performed recruitment in 3 stages: (1) We sent emails to a contact list of registered dietitians available on the RCD website, inviting them to take part in the research and answer the questionnaire; (2) in the same way, we sent invitations by direct messages to the professionals' Instagram accounts; and (3) professionals with more than 10,000 followers released the questionnaire link, inviting other dietitians to respond through the stories tool on Instagram*.*

### Data Collection

All participants in the study answered an online questionnaire about how they use social networks in their professions. The study data were collected and organized using REDCap electronic data capture tools hosted by the Federal University of Health Sciences of Porto Alegre [17,18].

To describe the use of social media, we based and adapted the questions included in the questionnaire through the Global Digital Report of 2021 [1]. We included additional questions to identify professionals' social network usage modes based on the study by Dunne et al [19]. We also created more questions to explore professionals' usage modes, opinions, and perceptions. We divided the questions into 6 categories: (1) sociodemographic information, (2) characterization of the sample, (3) negative and positive points perceived using social networks, (4) ways of using social networks, (5) professionals' perceptions on the use of social media, and (6) changes in perceptions and behavior on these platforms due to the COVID-19 pandemic.

### Data Analysis

Categorical results were presented by frequency and percentage. Symmetric quantitative results were presented by average and SD, or the median and the 25th percentile (P25) and the 75th percentile (P75) when asymmetric. Associations were analyzed using the chi-square test with the aid of adjusted standardized residues. The software used for the analyzes was SPSS Statistics v25 (IBM), and the significance level was .05.

## Results

### Participant Characteristics

In total, 334 dietitians were included in the study. Of the 334 dietitians who started, 289 (86.5%) participants reached the end of the questionnaire. Of these, 283 (97.9%) dietitians were female, and the median age was 29 (25-34) years. Information about the professional practice of the participants is shown in Table 1.

**Table 1:** Demographic and professional practice of dietitians who responded about their use of social networks (N=289), Brazil, 2021.

Characteristics		Value
**Demographics**		
	Gender (female), n (%)	283 (97.9)
	Age (years), median (P25-P75)	29 (25-34)
**Region, n (%)**		
	North	7 (2.4)
	Northeast	53 (18.3)
	Midwest	16 (5.5)
	Southeast	111 (38.4)
	South	102 (35.3)
Professional practice time (years), median (P25^a^-P75^b^)		5 (2-10)
**Professional field, n (%)**		
	Collective feeding nutrition	15 (5.2)
	Clinical nutrition	208 (72)
	Sports and exercise nutrition	34 (11.8)
	Public health nutrition	13 (4.5)
	Production chain: industry and food trade nutrition	4 (1.4)
	Teaching: research and extension nutrition	15 (5.2)

^a^P25: 25th percentile.

^b^P75: 75th percentile.

The use of social networks in the daily lives of dietitians was identified in 288 (99.7%) of the 289 participants, and of these, 264 (91.7%) claimed to use these platforms for their professional practice. Of the 24 (8.3%) professionals who stated that they did not use social networks linked to professional practice, 8 (33%) worked in the clinical nutrition field, 6 (25%) in public health nutrition, 5 (21%) in nutrition in collective feeding, and 5 (21%) in superior nutrition education and research. In a comparison of the number of dietitians who answered no to this question with the total number of professionals in the fields of professional activity, there were 8 (3.8%) of 208 in the clinical nutrition field, 6 (39%) of 13 in public health nutrition, 5 (33%) of 15 in nutrition in collective feeding, and 5 (33%) of 15 in superior nutrition education and research.

When evaluating modes of use of social networks by dietitians according to their working field, 133 (63.9%) of the 208 participants in the clinical nutrition field reported the habit of writing about topics such as food, feeding, and related ones, and 67 (32.2%) of these professionals reported the practice of recording videos on these subjects and sharing the videos on their social platforms. In contrast, 3 (20%) of the 15 professionals in the collective feeding field and 4 (21.1%) of the 19 professionals in the areas of superior nutrition education and research (n=15, 78.9%) and in the food industry (n=4, 21.1%) stated that they never wrote about these topics in their social networks. Regarding the videos related to these themes, 8 (53%) of the 15 nutrition professionals in the collective feeding field said they never recorded one, and 9 (47.4%) of the 19 nutrition professionals in the superior nutrition education and research field and in the food industry stated the same. The professionals in the clinical nutrition field stated the habit of following a schedule for organizing the content to be published on social networks. We detailed the use of these platforms in their professions as well as the modes of use in Tables 2 and 3, respectively.

**Table 2:** Characterization of the use of social networks by dietitians who responded about their use of social networks, Brazil, 2021.

Use of social networks		n (%)
Use of social networks in their daily lives (N=289)		288 (99.7)
Use of social networks related to professional practice (N=288)		264 (91.7)
**Most frequently used social network (N=264)**		
	Instagram	224 (84.8)
	WhatsApp	29 (11)
	Facebook	4 (1.5)
	LinkedIn	2 (0.8)
	Others	5 (1.9)
	Snapchat/Twitter	0
**Frequency of use of social networks (N=288)**		
	≥5 times a day	197 (68.4%)
	2-4 times a day	71 (24.7%)
	Once a day	10 (3.5%)
	3-6 times a week	9 (3.1%)
	<3 times a week	1 (0.3%)
**Time spent in organizing, creating, and posting materials (N=275)**		
	>6 hours per week	33 (12%)
	5-6 hours per week	38 (13.8%)
	3-4 hours per week	98 (35.6%)
	1-2 hours per week	59 (21.5%)
	<1 hour per week	23 (8.4%)
	I pay someone to do these tasks.	3 (1.1%)
	I do not do it.	21 (7.6%)

**Table 3:** Modes of use of social networks in professional practice by dietitians who responded about their use of social networks (N=288), Brazil, 2021.

Modes of use	Never, n (%)	Almost never, n (%)	Sometimes, n (%)	Almost always, n (%)	Always, n (%)
I share information about my services on social networks (eg, localization, specializations, and previous work).	17 (5.9)	24 (8.3)	61 (21.2)	72 (25)	114 (39.6)
I share third-party content related to health and food on my social media profiles.	31 (10.8)	81 (28.1)	123 (42.7)	33 (11.5)	20 (6.9)
I follow the work of other nutrition professionals through social networks.	2 (0.7)	4 (1.4)	46 (15.9)	64 (22.2)	172 (59.7)
I write about topics related to food/nutrition and share the content on my social networks.	13 (4.5)	18 (6.3)	38 (13.2)	53 (18.4)	166 (57.6)
I record food/nutrition videos and share them on my social networks.	66 (22.9)	38 (13.2)	60 (20.8)	45 (15.6)	79 (27.5)
Before sharing content on nutrition and health in general, I make sure that what I am posting is based on evidence and comes from a safe source.	2 (0.7)	3 (1.0)	9 (3.1)	19 (6.6)	255 (88.6)
Whenever I share any material, I make sure to credit the source.	15 (5.2)	28 (9.7)	58 (20.1)	52 (18.1)	135 (46.9)
I have an organized schedule for my social networks’ posts and content.	75 (26.0)	47 (16.3)	65 (22.6)	42 (14.6)	59 (20.5)

Dietitians identified the positive roles of social network usage in professional practice in the questionnaire. “I attract more clients/patients through social networks” was the most cited by 210 (72.7%) of the 289 participants, followed by “I keep in touch more easily with my clients” cited by 195 (67.5%) of the participants. Negative roles of social networks were also indicated, such as excessive time browsing social media, mentioned by 140 (48.4%) of the 289 participants. The second negative point, mentioned by 139 (48.1%) of the 289 professionals, was that the comparison with other professionals' accounts triggers a feeling of inferiority. Displayed in Multimedia Appendix 1 are all the data about the roles social networks play in the professional context of dietitians. Table 4 describes the professionals' general opinions and perceptions on the use of social media.

Regarding the changes in the use of social networks during the COVID-19 pandemic by dietitians, 217 (75.1%) of the 289 participants reported that they were performing online consultations according to the authorization given by the FCD (Brazil 2020). Of these 217, 179 (82.5%) professionals declared to be satisfied or very satisfied with this type of service. The changes related to feelings, beliefs, and behavior on the social networks of professionals during the COVID-19 pandemic are described in Table 5.

**Table 4:** Opinions and perceptions on the use of social networks by dietitians who responded about their use of social networks (N=289), Brazil, 2021.

Opinions and perceptions	Strongly disagree, n (%)	Somewhat disagree, n (%)	Neither agree nor disagree, n (%)	Somewhat agree, n (%)	Strongly agree, n (%)
Social networks are good tools to promote your services.	0	4 (1.4)	8 (2.8)	50 (17.3)	227 (78.5)
Licensed professionals are the ones who write most health-related content on social media.	80 (27.7)	108 (37.4)	33 (11.4)	60 (20.7)	8 (2.8)
Social networks play an important role regarding health promotion.	0	17 (5.9)	32 (11)	199 (41.2)	121 (41.9)
Being active on social media might be dangerous for professionals.	99 (34.2)	78 (20.1)	67 (23.2)	54 (18.7)	11 (3.8)
In social networks, most professionals behave according to the practical aspects established by the RCD^a^.	57 (19.7)	120 (41.5)	58 (20.1)	49 (17)	5 (1.7)
Social networks are unnecessary for dietitians.	210 (72.7)	31 (10.7)	19 (6.6)	15 (5.2)	14 (4.8)
Dietitians who post frequently on social media are at a commercial advantage when compared to those who do not post frequently or who do not have social networks accounts at all.	6 (2.1)	24 (8.3)	47 (16.2)	82 (28.4)	130 (45)

^a^RCD: Regional Council of Dietitians.

**Table 5:** Prevalence of behavior changes during the COVID-19 pandemic on social network usage related to the professional practice of dietitians who responded about their use of social networks (N=289), Brazil, 2021.

Behavior changes	n (%)
I noticed changes in my behavior, feelings, or beliefs regarding the use of social networks related to professional practice.	216 (74.7)
I started spending more time checking out the work of other nutrition professionals on social networks.	96 (33.2)
During the pandemic, I started to share or increased the frequency of sharing information about nutrition and health in general on social networks.	149 (51.6)
I felt more inspired to present information about my services on social networks.	127 (43.9)
I started using new tools (such as live video streaming, interactive question stickers, and polls, among others) on social networks.	125 (43.3)
During this period, I changed my belief regarding the relevance of using social networks for the professional practice of dietitians.	75 (26.0)
I felt some kind of concern for not feeling qualified enough to talk about subjects related to nutrition on social networks.	46 (15.9)

## Discussion

### Principal Findings

This study identified the roles that social networks play in the professional context of dietitians in Brazil and their modes of use, as well as the professionals' perceptions and opinions on the use of these tools and the changes in behavior on social network usage due to the COVID-19 pandemic. During this period, many professionals increased the frequency of their use of social media and changed their opinions on how relevant these tools are in their profession. The results showed that almost all the participants used social media for professional practice, which we expected since a large part of the population is already present on these platforms and uses them for professional reasons [1].

We identified that the use of social networks is more prevalent in clinical nutrition than in other fields. This result is relevant, as it shows there is a specific group of professionals who use these social platforms more frequently. It is possible to make a direct association between social networks and professional practice since internet users are making their occupations and activities public, sharing photographic records of their job duties, and searching for profiles that could be interested in their work. Therefore, we expected the association of social media with professional practice. Dietitians use these platforms to attract clients/patients, facilitate contact, and make it more frequent.

The large percentage of statements about using social networks to attract clients/patients is related to the fact that professionals post information about their services on these platforms, making it possible for the content to be visible to many internet users. As a positive point, professionals can quantify this number through metrics from the social networks themselves, allowing them to identify the numbers of views and interactions of posts. These metrics are not viable for offline media, which can be an advantage of social media compared to traditional media. Another almost exclusive feature of social networking, considered a positive point, is that internet users can contact professionals through direct and instant messaging.

Social networks also allow the professional to take an active role in the process of exchanging messages, identifying the profiles of potential clients/patients, and getting an opportunity to contact them. These actions are not viable in traditional media, such as banners, magazines, newspapers, or radio and television. Professionals can then use social media to write about topics they have mastered and spread that content to other users. When compiling information about a particular subject in their profile, other users start to perceive that professional as someone who shows authority on the subject, which brings credibility, thus facilitating more people from social networks to become interested in their services.

The participants in this study also pointed out the role of social networks as a means of facilitating contact with patients and making these contacts more frequent, which is relevant since professionals can closely monitor their patients/clients. Thus, the professional can send information at a faster speed and can use the tools to instruct their patients and answer questions. One of the advantages of social networks in this sense is that they have a wealth of visual tools that can facilitate the educational process. Sometimes, the professional can also opt for a more informal approach, which can influence the relationship with the patient. Still, social media has other types of tools that can make interactions between users more attractive and more frequent. The interactions provided by these platforms can also strengthen the bond between professional and patient. During the COVID-19 pandemic, a considerable part of the studied professionals reported starting using tools such as live video streams, polls, and question stickers on social networks, which may be related to the search for better interaction with the public, which was modified by the social distancing during the pandemic.

We expected the results about the indicated modes of use (sharing information about their services, checking the work of colleagues, and writing about topics such as food) since these behaviors are in line with the role social networks play in attracting clients/patients. Checking on colleagues' work was also expected since it is common for internet users to follow pages that are attractive to them, such as people who share common interests or colleagues with whom they previously had personal contact.

Still on the modes of use, the publication on topics related to food can bring credibility, as it provides “samples” of the professionals' knowledge. At the same time, attractive layouts or more casual content can also draw the attention of the social media public. We identified that publishing texts and videos on topics related to food is more frequent among professionals in the clinical nutrition field. In addition, the organization of content to be published using a schedule is an almost exclusive behavior of these professionals. In contrast, writing and recording videos on these topics is an uncommon behavior for professionals working in the areas such as collective deeding, superior nutrition education and research, and the food industry and commerce. These data suggest more frequent behaviors in a specific group of professionals within social networks, being less frequent in other areas. In this niche of work, there is a greater possibility of attracting clients, so these professionals dedicate more time to using social networks and have a variety of behaviors on these platforms.

Regarding the COVID-19 pandemic, some professionals reported adopting new behaviors on social networks or that certain existing behaviors have become more frequent. As an example, more than half of the participants stated that they started to publish or increased the frequency of publishing information about nutrition and health in general on their social networks. This was not surprising, since the social isolation measures adopted by the government in the country of study required more time indoors. When in isolation, it is expected that individuals will spend more hours of their day on the internet, including the use of social networks, since many activities started to occur virtually during the pandemic. It was expected that some professionals would start to explore these platforms as a possible way of extending their service, when prevented from conducting consultations and attracting clients in person. In addition, 2020, the year of isolation, was the year of the greatest growth for users of social networks in the past 3 years, which may have influenced the results mentioned earlier.

Most respondents cited the action of following the work of other professionals on social networks, which serves for dietitians to have references from other colleagues. Thus, it is possible to address topics the other professional discusses, becoming a source of ideas for their publications. Additionally, it can be useful to gather more knowledge about some subject that that colleague has mastered, so the interaction between professionals can be beneficial in these cases.

However, the frequent visit to other professionals' accounts can cause a feeling of inferiority, as reported as 1 of the negative points in using social networks by part of the dietitians participating in the study. Possibly, some facts caused this feeling, such as some professionals having more followers, appearing to be more successful than others, dominating specific subjects, or having a higher interaction rate in their accounts. Still, a third of the participants reported that they spent more time checking on the work of other professionals during the COVID-19 pandemic. This result could also be expected, considering the increase in the number of users and the time spent on social networks. Thus, it is reasonable that all reported behaviors grow in frequency, rather than just some of them exclusively.

Instagram was reported as the most used social network when linked to professional practice, which may be related to the platform's popularity in the country of the study. In Kemp [20]*,* Instagram was placed in the 11th position in the ranking of the most accessed websites and in 3rd place as an application in the category of social networks in the country, with an average time of use per user of 14 hours monthly in the mobile version.

In a similar study conducted in 2019 with sports dietitians in the United Kingdom [19], different results were found regarding the most used social networks in the professional practice of dietitians. Twitter was the most popular social network among participants, followed by Facebook and WhatsApp. In the year of the study (2019), Twitter had a higher number of users (46% of the UK population) compared to the current year (44.3%). Meanwhile, Instagram was 1 of the fastest-growing social networks in these years (from 47% of the population in 2019 to 52.5% in 2021) in the region [2,20]. The popularity of Twitter in the year of the study may be related to this result. Furthermore, 100% of the study participants had a Twitter account, while only 68% were present on Instagram. The percentage of professionals who used social platforms was high but lower than the results found in our study. This may be linked to the difference in percentages of the number of users and time spent on social networks in each region. In Brazil, it is estimated that 70.3% of the population are users of social media, with an average daily use of 3 hours and 42 minutes [20]; in the United Kingdom, in 2019, that number was around 67% of the population, and the average daily usage estimated was 1 hour and 50 minutes.

Most of the UK study participants pointed out that using social media is beneficial in their practice, which also occurred in our study. In the study mentioned, the ways of using these platforms were as follows: to update on scientific research, to search for recipes for meals, and to give information to their clients [19]. The results of our study showed that for the most part, professionals use social networks to share information about their services, to check on the work of professional colleagues, and to write about topics related to nutrition. The difference in the results of each study on the modes of use is notable, as it shows that social media can have different usage for professionals in the same field of activity. Thus, its use may vary according to the country and the social network used in each location.

An interesting result observed in this study was that the participants disagreed that accredited professionals are responsible for most of the content published on social networks, which corroborates with a systematic review that identified that issues of quality and reliability are 1 of the main limitations of the use of social media for communication in health [21]. Respondents also cited to believe that most professionals do not act according to the ethical aspects established by the RCD. As examples of violated behaviors in the Code of Ethics and Conduct of Dietitians, we can mention promotions or drawings of lots of procedures carried out by dietitians, dissemination of body images of themselves or third parties (even with authorization), and preference or promotion of products or company brands related to food and nutrition activities [22].

It is important to note that during the COVID-19 pandemic, some respondents reported they changed their views regarding the relevance of using social networks for their work practices. Still, a considerable part of the professionals started conducting online consultations with the permission of the FCD. The committee exceptionally suspended Article 36 of CFN Resolution No. 599, of February 25, 2018, which established that nutritional assessment and diagnosis should be carried out in person and that only nutritional guidance and monitoring could be conducted remotely. Thus, dietitians can carry out online consultations until the end of the COVID-19 pandemic [23,24], which shows the importance of the professionals' ability to adapt to the context in which they are inserted. Social networks became relevant for professionals when their reality changed, making these platforms a means to get closer to clients/patients.

A large part of the professionals participating in the study reported that they felt more motivated to present the services by digital means and that they changed their opinions and perceptions on social networks during the pandemic. The need to continue exercising their professional activities competitively and the inability to carry out face-to-face consultations may have contributed to a change in the way professionals perceive social networks and influenced their motivations to present their services on these platforms.

### Limitations

This study had limitations. Many of the responses to the questionnaire originated from invitations made through the Instagram platform itself, which reflected in the data of a greater number of users of this social network in our study. However, according to statistics on the use of social networks in Brazil, Instagram is 1 of the most popular ones [20], which also provides greater scope for researching this topic.

### Conclusion

In conclusion, this study explored the use of social networks by dietitians in Brazil and the changes in their use during the COVID-19 pandemic, identifying a wide adoption of these platforms for professional usage. Attracting clients and facilitating contact between professional and client were the main reported roles of social networks for dietitians. The modes of use reported by the professionals included sharing information about their services, following the work of professional colleagues, and writing about topics related to nutrition. Regarding the professionals' perceptions, most of them reported believing that social networks are an effective way to disseminate their services. Moreover, most professionals claimed to have noticed changes in their behaviors or beliefs on social media during the COVID-19 pandemic. Future research in this area could work with open questions to explore more in-depth different ways of using social media and what other benefits dietitians and other health professionals can obtain through them.

## Appendix

Multimedia Appendix 1 The roles social networks play in the professional context of dietitians who responded about their use of social networks (N=289), Brazil, 2021.

## Abbreviations

FCD: Federal Council of Dieticians
P25: 25th percentile
P75: 75th percentile
RCD: Regional Council of Dieticians
