# Supplementary material for: Use of Social Networks in the Context of the Dietitian’s Practice in Brazil and Changes During the COVID-19 Pandemic: Exploratory Study
Source: JMIR Form Res. 2022 Feb 25;6(2):e31533. doi: 10.2196/31533 (PMC8887558; doi:10.2196/31533)
Supplement: Multimedia Appendix 1 [file formative_v6i2e31533_app1.docx]

|  | **n (%)** |
| --- | --- |
| I attract more clients/patients through social networks | 210 (72,7%) |
| Through customer testimonials, I identify that I help people in making healthier choices when it comes to food | 167 (57,8%) |
| I easily update myself on new nutrition trends through social networks | 149 (51,6%) |
| I keep in touch more easily with my clients/patients through social networks | 195 (67,5%) |
| I exchange relevant information about nutrition with other professionals through social networks | 148 (51,2%) |
| I participate in health promotion campaigns and advocate relevant causes of the profession through social networks | 118 (40,8%) |
| I spend excessive time browsing social media | 140 (48,4%) |
| The comparison with other professionals' accounts triggers a feeling of inferiority | 139 (48.1%) |
| I feel demotivated that is caused by the low interaction on social networks | 135 (46,7%) |

**Multimedia Appendix 1.** The roles social networks play in the professional context of dietitians who responded about their use of social networks (N=289), Brazil, 2021.
